# Is Persistence a Hazard?

**DOI:** 10.1021/acs.est.6c00770

**Published:** 2026-03-23

**Authors:** Martin Scheringer, Ian T. Cousins

**Affiliations:** † Institute of Biogeochemistry and Pollutant Dynamics, ETH Zürich, 8092 Zürich, Switzerland; ‡ Department of Environmental Science, Stockholm University, SE-10691 Stockholm, Sweden

**Keywords:** environmental persistence, toxicity, exposure, regulatory science

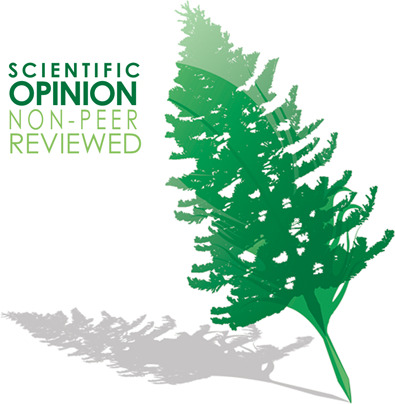

## Graphical abstract

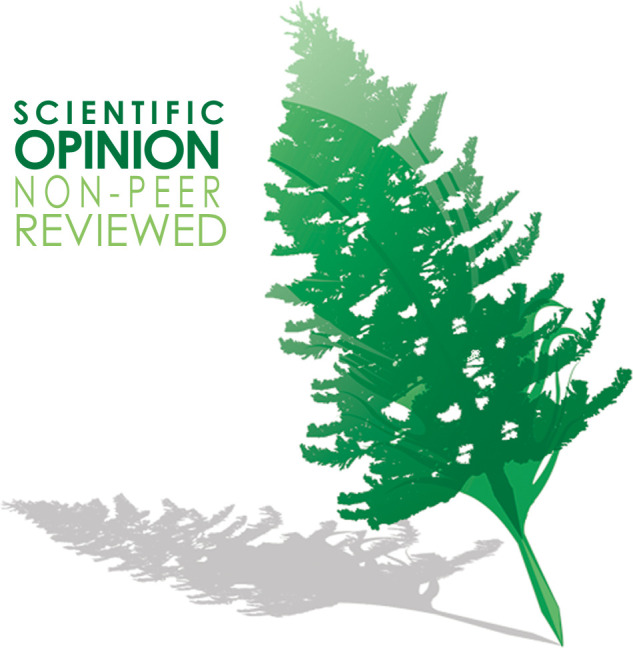

Why is the environmental persistence of chemicals so controversial in discussions about chemical hazard and risk? Why is it not generally accepted that environmental persistence is a hazard?

On the one hand, there have been (very) early warnings and clear empirical findings indicating serious problems caused by persistent chemicals exactly as a consequence of their environmental persistence.^1–3^ On the other hand, persistence is often framed as a chemical property “related to exposure” or “just indicating the presence of a chemical”, but not as a contributor to a chemical’s hazardousness. Attempts to put greater emphasis on environmental persistence as a hazard are often countered by statements such as, “sand, water, and nitrogen are persistent”, implying that persistence cannot be problematic. In this framing, toxicity is what matters, but only when exposure is relevant. Considering environmental persistence as a hazard does not fit the established scheme of risk assessment, which treats hazard as intrinsic toxic potency and exposure as something separate that can be measured, modeled, and controlled.

Here, we maintain that this conventional framing—the division of risk assessment into two “bins”, exposure versus intrinsic toxicity—obscures the meaning and importance of environmental persistence. In this scheme, all data on a chemical’s impacts must be assigned to one of these two bins. Exposure represents everything related to the presence of a chemical at a target site, and hazard represents the chemical’s toxic potency. Our point is that, within this perspective, there is no natural place for persistence, even though persistence inherently connects exposure and toxic potency, as we will outline below.

To clarify this, it is useful to recall what is meant by hazard in regulatory science. Hazard refers to an intrinsic property of a substance that has the potential to cause adverse effects, independent of how or where the substance is used. Persistence meets this definition because it is rooted in a chemical’s molecular structure and directly increases the magnitude, duration, and spatial scale of exposure. A highly persistent chemical therefore has a built-in potential to cause harm, even when its intrinsic toxic potency appears to be low.

It is straightforward that the effect-related properties of a chemical, all data showing adverse effects at levels from cells to organisms to populations, fit into the “effects” bin. However, exposure-related properties are more complicated because exposure depends on two very different factors: (i) the type, location, and strength of a chemical’s emission sources and (ii) the chemical’s environmental fate as an important “modifier” on the way from emission source to target. The first of these two, all aspects of a chemical’s use and emissions, is not a property of the chemical, i.e., is extrinsic to the chemical (does not derive from the chemical structure or properties of a substance). The second, environmental fate, includes phase partitioning, transport, and transformation/degradation, all of which are directly or indirectly affected by the chemical’s environmental persistence.

If a chemical is rapidly degraded, its environmental fate is not a major modifier of exposure on the way from the emission source to the target organism. The concentration at the target is determined by the distance to the source and the source strength. Exposure is high if the emission source is nearby and ends when emissions cease; exposure is low or nil if the emission source is far away and/or is no longer active. This is, however, different for persistent chemicals, for which environmental fate is characterized by long-term processes of multimedia partitioning, bioaccumulation, long-range transport, and slow degradation. In this case, exposure of a target organism is characterized by not only the chemical’s emissions source strength and the time and location of the emission sources but also, and even to a large extent, by the chemical’s low degradation rate constants, which lead to environmental persistence. Importantly, the chemical’s slow degradability is a direct feature of its chemical structure and, thereby, an intrinsic or inherent property. Exposure to highly persistent chemicals continues long after use and emissions end and is high even at locations far from the emission source.

In the two-bin perspective, persistence is generally assigned to the exposure bin, but the exposure bin is mostly seen as reflecting emission- and use-related aspects, not problematic inherent properties of a chemical. However, a chemical’s environmental persistence is exactly such a problematic inherent property. Like toxicity, it is independent of use pattern, use amounts, and emission source strength. Persistence adds to the detrimental effects of a chemical because it makes the exposure longer, higher, and more widespread. It therefore directly contributes to the occurrence of toxic effects. This is what makes persistence a hazardous property of a chemical.

The history of chemical pollution repeatedly demonstrates this. PCBs were initially considered safe because acute toxicity appeared low, yet their extreme persistence enabled global distribution, bioaccumulation, and late emerging effects on neurodevelopment and endocrine function. Well-studied PFASs followed a similar trajectory: low acute toxicity but high persistence, leading to worldwide contamination and chronic health effects that became apparent only after exposure for decades. These cases illustrate that persistence alone can transform modest toxic potency into large scale, long-term harm.

A further lesson from the history of persistent chemicals is that as knowledge about their toxicity improves, the levels once considered “safe” are repeatedly revised downward. This pattern has been observed across multiple classes of persistent substances, with PFASs being a particularly striking case. The drinking water guideline for PFOA in the state of West Virginia was 150 μg/L in 2002,^4^ whereas in 2025, the U.S. EPA announced final national primary drinking water regulations for PFOA and PFOS of 4 ng/L.^5^ This is a decrease by a factor of 37 500. Notably, the maximum contaminant level goals (MCLGs) for PFOA and PFOS were set at zero in the same rule. Such continual downward revision indicates that traditional risk assessment cannot reliably protect against long-term harm. Risk assessment implicitly assumes that hazards are well-characterized, that threshold values are stable over time, and that exposures can be controlled through management measures. For highly persistent substances, none of these assumptions hold.^6^ Toxicity is often underestimated for decades; exposure is effectively irreversible, and contamination accumulates globally long before risks are fully understood. By the time risk assessment catches up with emerging evidence, widespread and long-lasting harm has already occurred. This dynamic underscores why persistence must be treated as a hazard in its own right rather than as a factor that can be managed within traditional risk-based frameworks.

These recurring patterns, long-term global impacts and continual downward revision of “safe” levels, show that the concern about a chemical’s impacts is not about persistence per se, but about persistence in combination with adverse effects. Nearly all chemicals—here we refer to organic, nonpolymeric chemicals—have some toxicity because they are taken up by cells, circulate in the bloodstream, interact with receptors, and thereby cause adverse effects. Their toxic potency varies over many orders of magnitude, but “the dose makes the poison” implies that at some dose level adverse effects occur even for chemicals of relatively low toxicity. High persistence is a key driver of increasing doses and long-term exposures, increasing the likelihood that larger fractions of the population will eventually reach doses at which adverse effects manifest.

Because persistence leads to continuous, sometimes lifelong exposure, it increases the likelihood that chronic diseases will emerge over time, as illustrated by the growing body of evidence linking PFAS exposure to cancers, metabolic disorders, immune dysfunction, and other long-latency outcomes. These conditions are particularly burdensome because they develop slowly, are difficult to diagnose early, and impose substantial long-term costs on individuals and healthcare systems. Persistence therefore does not merely increase exposure; it amplifies the societal and medical consequences of any underlying toxicity by ensuring that large populations remain exposed for long periods of time.

Recognizing persistence as a hazard implies that persistent chemicals are inherently unsafe regardless of current toxicity data, since their long-term, irreversible accumulation creates risks that cannot be controlled.^6^ This conclusion challenges established regulatory frameworks and economic interests, which is why persistence is still not universally accepted as a hazard.

Recognizing persistence as a hazard also points toward the need for designing chemicals so that they do not remain in the environment for long periods of time. Following Principle 10 of the Principles of Green Chemistry (“Design for Degradation”),^7^ substances should be engineered to degrade into harmless products once their intended function has been fulfilled. In practice, however, persistence is often deliberately built into chemical products to provide durability, stability, and long service life, which are important properties in many applications. This creates a genuine design challenge of how to generate the necessary function and performance in products while avoiding environmental persistence. Addressing this challenge requires functional substitution, novel product and process designs that work without releases of persistent chemicals to the environment, and regulatory incentives that discourage the creation of new persistent “novel entities”.
